# Ultrafine FeNi_3_ Nanocrystals Embedded in 3D Honeycomb-Like Carbon Matrix for High-Performance Microwave Absorption

**DOI:** 10.3390/nano10040598

**Published:** 2020-03-25

**Authors:** Congai Han, Haiyan Zhang, Danfeng Zhang, Yunfei Deng, Junyao Shen, Guoxun Zeng

**Affiliations:** 1 School of Material and Energy, Guangdong University of Technology, Guangzhou 510006, China; hca0109gdut@163.com (C.H.); dyf1012gdut@163.com (Y.D.); Junyao-Shen@gdut.edu.cn (J.S.); zenggx@gdut.edu.cn (G.Z.); 2 School of Computer Science and Technology, Guangdong University of Technology, Guangzhou 510006, China; dfzhang@gdut.edu.cn

**Keywords:** FeNi_3_@C composites, 3D honeycomb-like carbon, microwave absorption

## Abstract

The reasonable design of magnetic carbon-based composites is of great significance to improving the microwave absorption (MA) performance of the absorber. In this work, ultrafine FeNi_3_ nanocrystals (5–7 nm) embedded in a 3D honeycomb-like carbon matrix (FeNi_3_@C) were synthesized via a facile strategy that included a drying and carbonization process. Because of the soft magnetic property of the FeNi_3_ nanocrystals and their unique 3D honeycomb-like structure, the FeNi_3_@C composites exhibit excellent MA abilities. When the filler loading ratio of FeNi_3_@C/paraffin composites is only 30 wt%, the maximum reflection loss (RL) value is −40.6 dB at 10.04 GHz. Meanwhile, an ultra-wide absorption frequency bandwidth of 13.0 GHz (5.0–18.0 GHz over −10 dB) can be obtained in the thickness range of 2.0–4.5 mm, and this means that the absorber can consume 90% of the incident waves. It benefits from the dual loss components, multiple polarizations, and multiple reflections for improving MA performances of FeNi_3_@C composites. These observations suggest that the 3D honeycomb-like FeNi_3_@C composites have broad application prospects in exploring new MA materials that have a wide frequency bandwidth and strong absorption.

## 1. Introduction

In recent years, a wide usage of electronic equipment has caused more and more electromagnetic pollution [1,2,3]. Long-term exposure to this pollution will cause specific harm to human health, and will also interfere with the operation of sensitive electronic equipment in civil and military fields [4].Thus, microwave absorption (MA) materials are urgently needed to attenuate superfluous electromagnetic (EM) energies [5,6]. The MA materials effectively convert incident EM microwaves energy into thermal energy or dissipate them via interference [4,7,8]. To date, a range of traditional MA materials (including dielectric materials, such as graphene, carbon nanotubes, carbon fiber, and ZnO, and magnetic materials, such as Fe, Co, Ni, Fe_2_O_3_, and Fe_3_O_4_) have been widely studied for their MA performance [4,9,10,11]. However, it is difficult to achieve excellent MA properties for a single loss component, because these traditional absorbers have the drawbacks of thick thickness, weak absorption, and narrow absorption bandwidth. Therefore, exploring novel microwave absorbers is highly needed. More and more researchers think highly of nanoscale soft magnetic materials and their alloys because they own low coercive forces, high saturation magnetization, high Curie temperatures, and a high Snoek’s limit at high frequency bands. FeNi_3_ alloy, as a typical soft magnetic material, has aroused great interest from researchers, due to its superior magnetic properties.

Accordingly, a simple and effective way to improve MA performance is to combine dual loss (magnetic and dielectric loss) components into composite materials using a well-designed method [12]. Che et al. [13] filled Fe into carbon nanotubes (CNTs) via a simple catalytic pyrolysis routine, and the composite material showed excellent microwave absorbability. Cao et al. [14] introduced multi-walled carbon nanotubes (MWCNTs)/Fe_3_O_4_ nanocrystals composites for EM microwaves absorbers via a co-precipitation way. MA properties of the composites are better than those of pure MWCNTs and other Fe_3_O_4_ hybrid materials. Thus, many composites such as Ni/C porous nanofibers [15], Co@C spheres [16], FeCo-C core-shell nanoparticles [17], Fe_3_O_4_/CNTs [18], and C@NiCo_2_O_4_@Fe_3_O_4_ [19], have been explored for microwave absorption. Normally, both the compounds in the composites as well as the microstructure affect the MA properties. As a result, numerous morphologies have been successfully prepared to attain strong microwave attenuation ability. Liu and coworkers [20] designed flower-like Co_20_Ni_80_ alloy spheres with an absorption bandwidth of 5.5 GHz and an urchin-like Co_20_Ni_80_ alloy with a reflection loss (RL) value of −33.5 dB. Shen et al. [21] prepared Co-C nanofibers via electrospinning, and the nanofibers had an optimal RL value of −33.1 dB at 14.1 GHz. Zhao et al. [22] investigated the MA properties of 2D sponge-like Ni/derivative heterostructures, and they found that the value of maximum RL was −37.3 dB at 7.1 GHz when the thickness was 3.3 mm. As above, substantial improvements to the MA performance were achieved with various morphological structures. However, there is still a long way to go to optimize MA composites with a consideration of their microstructure and composition at the same time.

In our work, we successfully synthesized 3D honeycomb-like FeNi_3_@C composites using a facile strategy. As-prepared FeNi_3_ nanocrystals are uniformly surrounded by a carbon matrix that protects the FeNi_3_ nanocrystals from agglomeration. The FeNi_3_@C composites benefit from the soft magnetic property of the FeNi_3_ nanoparticles and unique 3D honeycomb-like structure, and thus they exhibit excellent MA abilities. The synergy effects, concluding dielectric/electrical losses and magnetic loss, efficiently improve the MA performance. The 3D honeycomb-like FeNi_3_@C composites obtain the maximum RL value of −40.6 dB and a broad frequency bandwidth of 13.0 GHz. This work provides a facile approach for the synthesis of a magnetic alloys/carbon composites absorber with wide bandwidth and strong absorption.

## 2. Materials and Methods

### 2.1. Materials

Sodium lauryl sulfate (SDS), styrene (C_8_H_8_), divinylbenzene (DVB), potassium persulfate (K_2_S_2_O_8_), tetrahydrofuran (THF), polyvinylidene fluoride (PVDF), bis (η5-2, 4-cyclopentadien-1-yl) nickel (C_10_H_10_Ni), and ferrocene (C_10_H_10_Fe) were acquired from Aladdin Industrial Co, Shanghai. All chemical reagents used in reaction are analytical grade and without further treatment.

### 2.2. Preparation of Polystyrene (PS) Spheres Emulsion Solution

An emulsion solution of PS spheres was prepared via a typical emulsion polymerization method. The specific procedure was described as follows: 240 mg SDS was completely dissolved in 1200 mL distilled water with a 30-minute stirring. Then, 30 mL C_8_H_8_ and 3.6 mL DVB were added under continuous stirring. Subsequently, the solution was heated to 75 °C under a N_2_ atmosphere, with 1.2 g K_2_S_2_O_8_ adding. After 3 h, 3.6 mL DVB was added to the mixed solution system again. Finally, the PS emulsion solution was formed and stored for later use.

### 2.3. Synthesis of FeNi3@C Composites

The as-papered PS emulsion solution was dried overnight at 80 °C under vacuum to obtain a powder of the PS spheres. Ultrasonication was used to disperse 0.15 g of the powder of PS spheres in 20 mL of THF. Then, 1.00 g C_10_H_10_Ni, 0.33 g C_10_H_10_Fe and *x* g of PVDF (*x* = 0.3, 0.15, and 0.075) were added to the mixed solution with a 2-h stirring. The mixtures were then transferred into a homemade oven to de-solvate overnight under heat at 80 °C. After removing the solvent (THF), the remaining precursors were calcined at 600 °C for 2 h under an Ar/H_2_ atmosphere; the heating rate was 5 °C min^−1^. The obtained FeNi_3_@C composites were labeled as S1 (0.30 g PVDF), S2 (0.15 g PVDF), and S3 (0.075 g PVDF).

### 2.4. Preparation of the Coaxial Sample

**Figure 1:**
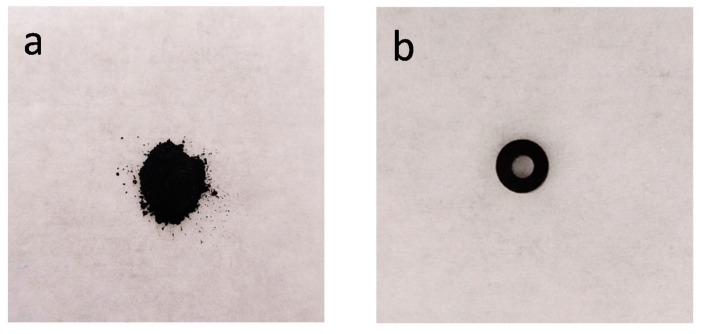
(**a**) The photo of FeNi_3_@C pristine powder. (**b**) The photo of FeNi_3_@C/paraffin ring-shaped sample.

The FeNi_3_@C composites and paraffin were mixed in a certain ratio and heated at 80 °C for 10 min. Then the system was cooled to room temperature and pressed into a ring-shaped sample ($\varphi_{in}=3.04\;\mathrm{mm}$, $\varphi_{out\;}=7.00\;\mathrm{mm}$) under a pressure of 1.5 MPa. The photos of pristine powder and ring-shaped sample can be seen in Figure 1.

### 2.5. Characterizations

The phase and component information of the composites were characterized using an X-ray diffraction instrument (XRD, Germany, D8 ADVANCE). Both field emission scanning electron microscopy (FESEM, Hitachi, SU8220) and high-magnification transmission electron microscopy (HRTEM, FEI, TALOS F200S) were performed to observe the morphology of the product. Energy dispersive spectrometry (EDS), as an accessory of FESEM, was used to detect the distribution elements. The operation voltage was 20 KV for FESEM and 200 KV for HRTEM. Thermogravimetric analysis (TGA) was performed from room temperature to 900 °C with a heating rate of 10 °C min^−1^ on a TGA instrument. The degree of graphitization of the products was analyzed using Raman spectroscopy with an excitation wavelength of 532 nm (HORIBA Jobin Yvon, France, LabRAM HR Evolution). X-ray photoelectron spectroscopy (XPS) was used to investigate the chemical states and compositions of the composites. Vibrating sample magnetometry (VSM) was carried out to survey the magnetic properties of the FeNi_3_@C composites. An AV3618 network analyzer was used for coaxial measurements of the electromagnetic parameters in the frequency range of 2–18 GHz.

## 3. Results and Discussion

**Figure 2:**
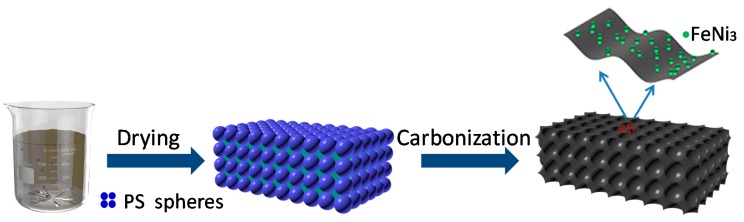
Schematic illustration of the synthesis of 3D honeycomb-like FeNi3@C composites.

Figure 2 is a schematic illustration of the whole preparation process of the 3D honeycomb-like FeNi_3_@C composites. The PS spheres were synthesized via an emulsion polymerization method, and then a precursor template was used. The PS spheres, C_10_H_10_Fe, C_10_H_10_Ni, and PVDF were added to the THF solvent; the mixture was then sequentially dried and carbonized. PVDF serves as a carbon source and a binder to connect the spheres during the drying process, and this causes the metal cations to be evenly distributed in the space of spheres. During the final carbonization process, PS spheres were removed to produce nanoscale pores. Simultaneously, PVDF decomposed and formed a carbon matrix. With prolonged heating, the Fe/Ni-based oxides that were produced during the early stage were reduced by H_2_ to the corresponding metals [23,24]. The reaction of intermetallic FeNi_3_ can be simply described as follows [25]: Fe + 3Ni→FeNi_3_.

**Figure 3:**
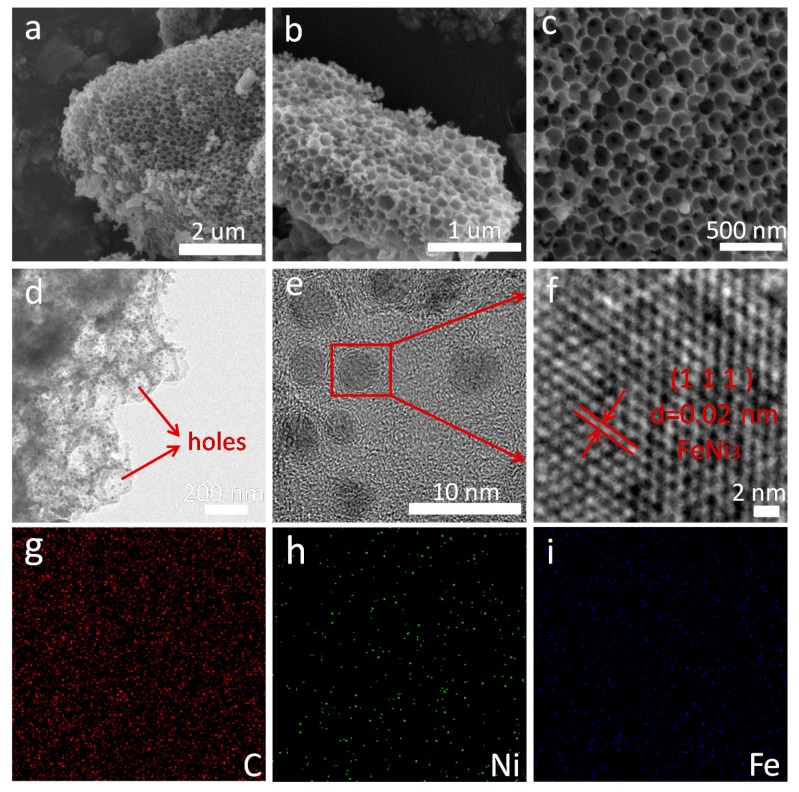
SEM images of S2 at (**a**) low, (**b**) medium, and (**c**) high magnification. (**d**–**f**) TEM images of S2 at different resolutions. EDS mapping images of (**g**) C, (**h**) Ni, and (**i**) Fe elements.

Scanning electron microscopy (SEM) and HRTEM were used to assess the microstructure of the as-prepared FeNi_3_@C product. Notably, all the samples showed a 3D honeycomb-like structure with no obvious differences (Figure S1a–d). Therefore, taking S2 sample as an example, the microstructure analysis is discussed. As shown in Figure 3a–c, nanopores are distributed uniformly and in an orderly manner in the carbon matrix under different magnifications. The average size of the pores is about 130–150 nm, with mainly controlling from the PS spheres. There is no doubt that this unique porous structure will enhance the scattering of EM waves, thus facilitating the enhancement of MA properties, which have been proved [10]. Figure 3d,e represent typical TEM images of sample S2, in which the FeNi_3_ nanocrystals are well coated by a honeycomb-like carbon layer. The ultrafine nanocrystals are sphere-like and the average size of particles is 5–7 nm. In Figure 3f the periodic fringe spacing is 0.02 nm, which can be assigned to the (111) plane of FeNi_3_. Figure 3g–i shows EDS mapping of S2. The results indicate that Fe, Ni, and C were well distributed, and this is in good agreement with the XPS analysis.

**Figure 4:**
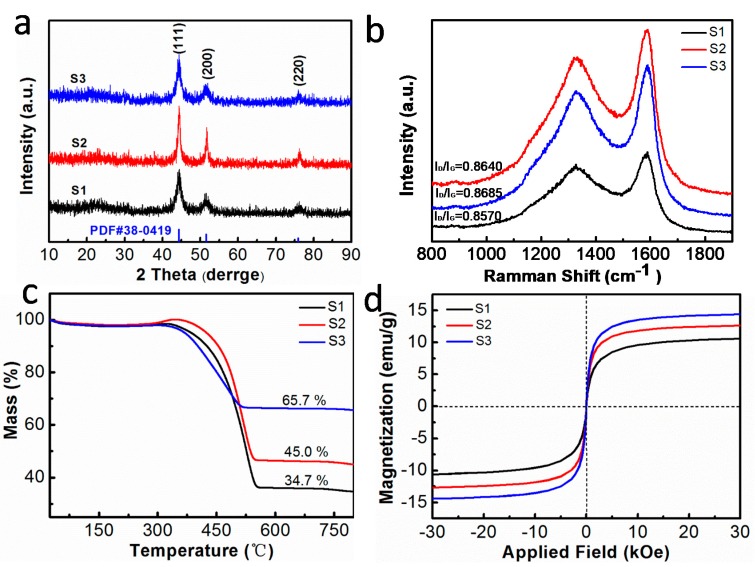
(**a**) XRD patterns of the S1, S2, and S3 samples. (**b**) Raman spectra of the S1, S2, and S3 samples. (**c**) Thermogravimetric (TG) curves of all of the samples under an atmosphere of air. (**d**) Hysteresis loops of the S1, S2, and S3 samples.

Figure 4a shows the XRD patterns of all of samples. All samples are highly crystalized with three diffraction peaks. The peaks located at 44.28°, 51.53°, and 75.87°, respectively, correspond to the (111), (200), and (220) planes of FeNi_3_ (PDF#38-0419) with face-centered cubic (FCC) structures. Our products are free of the oxidation disruption because no peaks of Fe/Ni-based oxides can be found in the composite. Considering the absence of graphitization peaks, the honeycomb carbon matrix is considered as an amorphous structure, which is consistent with the results of EDS and TEM. Raman spectra (Figure 4b) were further studied to examine the presence and crystallinity of carbon matrix. There are two prominent peaks at 1333 cm^−1^ and 1590 cm^−1^, and these respectively correspond to the D and G bands. Generally speaking, the D band of the A_1g_ vibrational mode is considered to represent sp^3^ carbon atoms in disordered graphite, and the G band of the E_2g_ vibrational mode of the C-C bond stretching represents sp^2^ carbon atoms in a 2D hexagonal lattice [26,27]. The value of the I_D_/I_G_ ratio can be used to evaluate the degree of disorder in the FeNi_3_@C composites and indicates the presence of structural defects. Furthermore, these defects facilitate the polarization of dipole and thus improve the microwave absorption performance, as reported by Ding [26]. The I_D_/I_G_ values of S1, S2, and S3 are 0.8570, 0.8640, and 0.8685, respectively. Obviously, the I_D_/I_G_ values of S1, S2, and S3 show an increasing trend, and this can be attributed to a decrease in the amount of added PVDF, which has a lower degree of graphitization.

As shown in Figure 4c, the thermal gravimetric curves of all of the FeNi_3_@C samples present two weight loss stages. One stage (below 200 °C) is caused by the removal of surface-absorbed water. The second stage (between 320 °C and 530 °C) corresponds to the combustion of the carbon layer. The final products are FeNi_3_ nanocrystals with Fe/Ni-based oxides and the values of the residual weight of S1, S2, and S3 are about 34.7%, 45.0%, and 65.7%, respectively. In this experiment, PVDF is used as a carbon source, and different amount of PVDF means the different content of carbon source. The carbon content of three samples is reduced in a high temperature oxidation process, resulting in different thermal weight loss. The residual weight values of S1, S2, and S3 show an increasing trend and this can be attributed to a decrease in the amount of added carbon source.

Figure 4d shows hysteresis loops for all samples measured using VSM at room temperature. Because the FeNi_3_ nanoalloy is a kind of soft magnetic material, S1, S2, and S3 all exhibit typical soft magnetic behavior [26]. The values of the saturation magnetization (Ms) of S1, S2, and S3 are 10.6, 12.6, and 14.4 emu/g, respectively. Relative coercivity (Hc) values are 49.6, 50.1, and 50.3 Oe, respectively. Ms of S1 is the lowest among that of the three samples due to its high content of the carbon source. We found that the Ms value of FeNi_3_@C is significantly lower than the Ms value of pure FeNi_3_ (Ms = 85.2 emu/g) [28]. The decrease Ms can be ascribed to the presence of nonmagnetic carbon materials. Furthermore, the 3D honeycomb-like structure that has more defects in the crystalline structure may cause surface imperfections in the FeNi_3_@C composites. Other affects for losing Ms comes from the oxidation vulnerability and nanoscale lattice mismatch in ultrafine FiNi_3_ nanocrystals. This thus leads to a much smaller Ms value [29,30,31]. Additionally, the grain size and shape anisotropy influence the Hc values of magnetic materials [32]. The higher coercivity of FeNi_3_@C composites may be due to the small grain size of FeNi_3_ nanocrystals.

**Figure 5:**
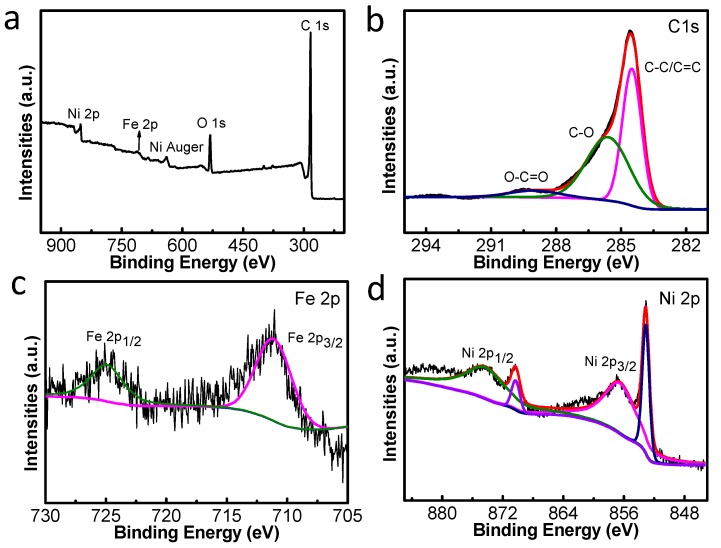
X-ray photoelectron spectroscopy (XPS) spectra of the sample of S2: (**a**) survey scan of FeNi_3_@C composites; (**b**) C 1s spectrum; (**c**) Fe 2p spectrum; (**d**) Ni 2p spectrum.

The XPS measurements were used for further analyzing the surface chemical compositions and elemental valence states of the samples. The presence of Fe, Ni, and C in the synthesized FeNi_3_@C composites was confirmed by the spectrum of the survey scan (Figure 5a). The survey scan shows five peaks with binding energies of approximately 285, 531, 641, 712, and 856 eV, and these are assigned to C 1s, O 1s, Ni Auger, Fe 2p, and Ni 2p, respectively. These indicate the presence of C, O, Fe, and Ni in the sample. The existence of oxygen may be due to the fact that the original material PVDF contains some oxygen-containing functional groups [33]. However, we did not find oxygen element in the EDS mapping, indicating that the oxygen content in the sample is extremely low. The C1s spectrum (Figure 5b) can be split into three peaks that are located at 284.6, 286.3, and 288.8, and these correspond to C − C/C = C, C − O, and O − C = O, respectively. Figure 5c displays the Fe 2p spectrum of S2, and it can be fitted by two peaks. One peak at 711.6 eV corresponds to Fe 2p_3/2_ and one peak at 724.5 eV corresponds to Fe 2p_1/2_. As seen in Figure 5d, the fitting peak with binding energies of 856.5 and 874.3 eV should be assigned to Ni 2p_3/2_ and Ni 2p_1/2_, respectively. Also, the peaks at 853.1 and 870.3 eV can be attributed to the Ni 2p_3/2_ and Ni 2p_1/2_ of Ni^2+^ states, respectively [4].

**Figure 6:**
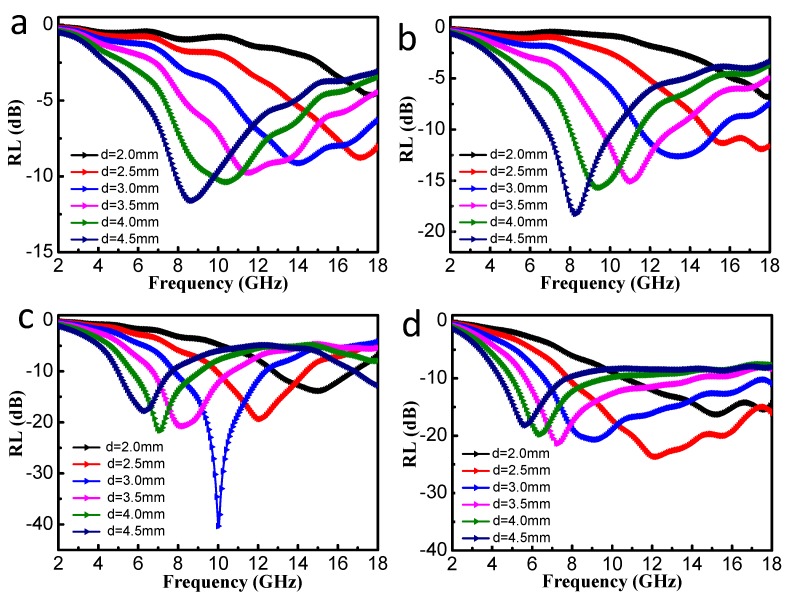
Calculated reflection loss (RL) curves of the S2–paraffin composites with different absorber thicknesses: (**a**) S2–10 wt%, (**b**) S2–20 wt%, (**c**) S2–30 wt%, and (**d**) S2–40 wt%.

In order to explore the influence of the filler loading ratios on the MA performance, we took the S2 sample as an example to measure the electromagnetic parameters. The content of S2 in the paraffin matrix varied from 10 wt% to 40 wt%, and was labeled as S2–10 wt%, S2–20 wt%, S2–30 wt% and S2–40 wt%, respectively. In Figure 6c, when the thickness is 3.0 mm, the maximum RL value of S2–30 wt% can reach −40.6 dB. However, for layers of the same thickness, the maximum RL values of S2–10 wt%, S2–20 wt%, and S2–40wt% are −9.13 dB, −12.62 dB, and −20.68 dB, respectively. Obviously, S2–30 wt% has the largest RL value at the same thickness. Therefore, the 30% filler loading ration was chosen for comparing the MA properties of other samples in the following discussion.

RL values are usually used to assess the performance of MA materials. These values can be calculated according to transmission line theory, and the equations are as follows [2,34]:

(1) $$\mathrm{RL}\left(\mathrm{dB}\right)=20\;\log_{10}\left|\frac{Z_{in}-Z_{0}}{Z_{in}+Z_{0}}\right|$$

(2) $$Z_{in}=Z_{0}\sqrt{\mu_{r}/\varepsilon_{r}}\tanh\left[j\left(2\pi fd/c\right)\times \sqrt{\mu_{r}\times \varepsilon_{r}}\;\right]$$

where $Z_{in}$ represents the input impedance at the surface of the absorber, $Z_{0}$ is the impedance of free space, d is thickness of the sample, f is the frequency of the electromagnetic wave, and c is the speed of light. When the RL values are −10 and −20 dB, it means that 90% and 99% of the incident EM waves are attenuated [35,36]. Also, the frequency ranges of RL values below −10 dB are valuable for engineering applications [10].

**Figure 7:**
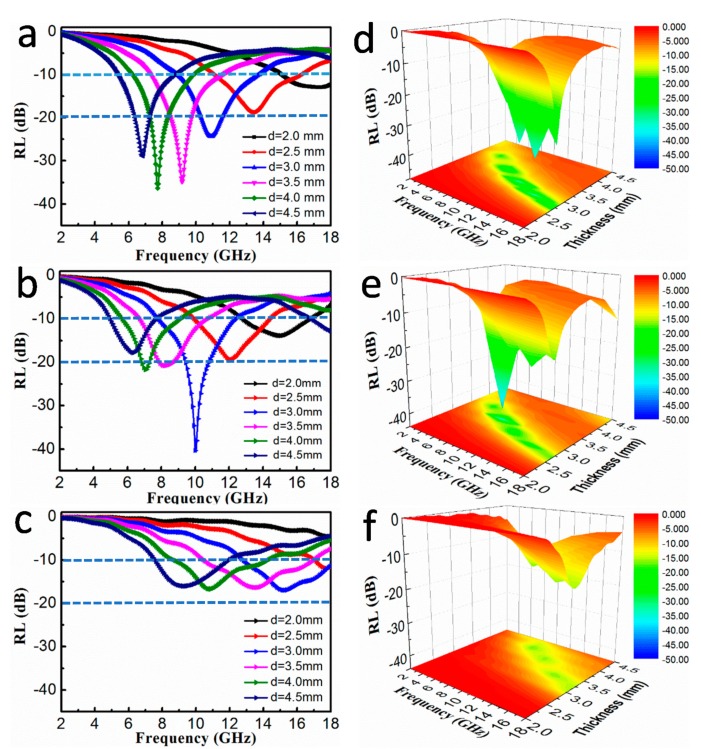
Frequency dependence of RL curves at different thicknesses of (**a**) S1, (**b**) S2, and (**c**) S3 with a filler loading ratio of 30 wt%. Three-dimensional diagrams of RL values for (**d**) S1, (**e**) S2, and (**f**) S3.

Figure 7 presents RL curves for S1, S2, and S3 at various thicknesses, each with filler loadings of 30 wt%. It is clear that with an increase in thickness, the RL peaks move toward the lower frequency region, and this is consistent with other reports [2,37]. All samples are consistent with quarter-wavelength matching model, where the relationship between sample thickness (d) and absorption peak frequency (f) can be expressed as the following equation [38]:

(3) $$d\;=nc/4f\sqrt{\left|\varepsilon_{r}\right|\times \left|\mu_{r}\right|}\left(n=1,2,\cdots \right)$$

When d and *f* satisfy this formula, the value of RL can be calculated. As seen in Figure 7a, the maximum RL value of S1 can reach −36.43 dB at 7.72 GHz when the thickness is 4.0 mm. In Figure 7c, the RL value of S3 is only −16.35 dB at 13.56 GHz when the thickness is 3.5 mm. In addition, the S2 possess the maximum RL value of −40.6 dB at 10.04 GHz and an ultra-wide bandwidth (RL ≤ −10 dB) of 13.0 GHz in the range of 5.0–18.0 GHz, which we can see from Figure 7b. This suggests that the S2 exhibits more excellent MA performance compare to S1 and S3. To better assess the MA properties, Figure 7d–f presents the 3D RL diagram of all the FeNi_3_@C samples with different thicknesses. Furthermore, the RL values calculated at a thickness of 3.0 mm are shown in Figure S3. The maximum RL value first decreases and then increases as the carbon source decreases. As a result, the S2 sample with 0.15 g of added PVDF has the optimal MA performance. This can be attributed to the gradual formation of a conductive network that occurs when the content of the carbon source increases, and this results in conductive losses that dissipate incident EM waves [22]. However, high conductivity may lead to poor MA properties when the filler loading ratio of the carbon source is increasing. These results suggest that the amount of added PVDF had a remarkable effect on the MA properties of the absorber, and thus, the amount of added PVDF should be well optimized.

It is known that, the MA properties of an absorber are highly dependent on the relative complex permittivity ($\varepsilon_{r}=\varepsilon^{\prime }-j\varepsilon^{\prime \prime }$) and relative complex permeability ($\mu_{r}=\mu^{\prime }-j\mu^{\prime \prime })$. Generally, the real parts ($\varepsilon^{\prime }$ and $\mu^{\prime }$) and imaginary parts ($\varepsilon^{\prime \prime }$ and $\mu^{\prime \prime }$) represent the storage and dissipation capabilities of electric and magnetic energy, respectively [39]. The dielectric loss tangents ($\tan\delta_{\varepsilon }=\varepsilon^{\prime \prime }/\varepsilon^{\prime }$) and magnetic loss tangents ($\tan\delta_{\mu }=\mu^{\prime \prime }/\mu^{\prime }$) are widely used to evaluate the dielectric loss capability and magnetic loss capability of the absorber.

**Figure 8:**
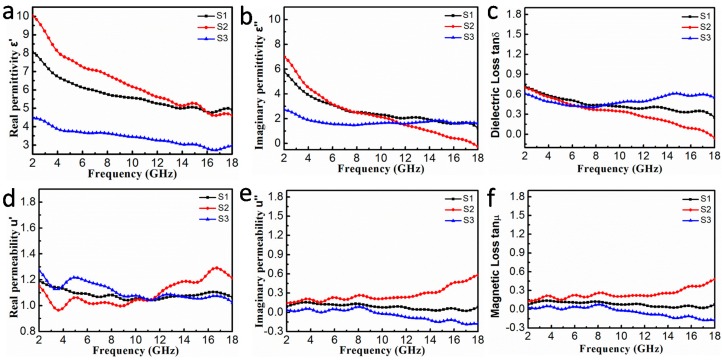
(**a**) Real and (**b**) imaginary parts of complex permittivity for S1, S2, and S3. (**d**) Real and (**e**) imaginary parts of complex permeability for S1, S2, and S3. (**c**) Dielectric loss tangents and (**f**) magnetic loss tangents of S1, S2, and S3.

Figure 8 displays vital parameters ($\varepsilon^{\prime },$ $\varepsilon^{\prime \prime },\;\mu^{\prime },\;$ and $\mu^{\prime \prime }$) for all of the samples. In Figure 8a, it is obvious that with the increase of PVDF loading, the values of the real part of the relative complex permittivity first increases and then decreases. Also, typical frequency dispersion behavior for all of the samples is shown in the Figure 8a,b) [32]. Over the entire frequency range, the values of $\varepsilon^{\prime }$ and $\varepsilon^{\prime \prime }$ for S2 dropped from 10.02 to 4.61 and 6.70 to 0.27, respectively, with slight fluctuates, and a similar decrease is observed in S1 and S3. Liu et al. [35] found that moderate complex permittivity is more favorable for the MA performances of an absorber than a lower or higher complex permittivity. This is consistent with the high microwave absorption properties of S2, as reported in our study. The dielectric loss factor is plotted versus frequency (Figure 8c), and a higher value of $\tan\delta_{\varepsilon }$ indicates a higher ability to dissipate the microwave energy. In alternating electromagnetic fields, there are different variations in $\tan\delta_{\varepsilon }$ and these indicate that multiple polarization relaxation processes occurring in the FeNi_3_@C composites.

**Figure 9:**
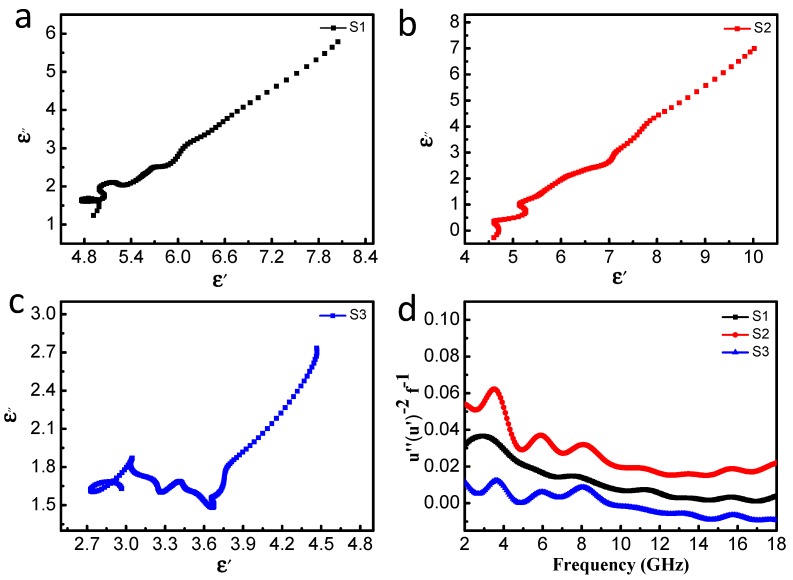
Curves of $\varepsilon^{\prime }$ versus $\varepsilon^{\prime \prime }$ (Cole–Cole semicircles) for (**a**) S1, (**b**) S2, and (**c**) S3. (**d**) Frequency dependences of $\mu^{\prime \prime }{\left(\mu^{\prime }\right)}^{-2}f^{-1}$ values for S1–S3.

Generally, the dielectric loss capability mainly derives from conductivity loss and polarization relaxation behaviors according to free electron theory and Debye theory [40,41]. Conductivity loss may result from the conductive network, which is caused by the 3D honeycomb-like structure. Also polarization relaxation can be divided into four polarization modes: electronic, ionic, dipole orientation, and interfacial polarization [32,42]. Ionic polarization and electronic polarization are not considered in our study because they usually occur in the high frequency range of 10^3^–10^6^ GHz [42]. Thus, the dielectric loss mainly originates from interfacial polarization and dipolar polarization, and these are caused by defects during carbonization. Rich interfaces between FeNi_3_ cores and the graphite layer as well as between the graphite layer and paraffin lead to sufficient interfacial polarization for dielectric loss [32]. To further prove the diversified dielectric loss mechanism, curves of $\varepsilon^{\prime \prime }$ versus $\varepsilon^{\prime }$ were plotted, as shown in Figure 9. According to Debye theory, $\varepsilon^{\prime \prime }$ and $\varepsilon^{\prime }$ meet the formula [12,43]:

(4) $${\left(\varepsilon^{\prime }-\frac{\varepsilon_{s}+\varepsilon_{\infty }}{2}\right)}^{2}+{\left(\varepsilon^{\prime \prime }\right)}^{2}={\left(\frac{\varepsilon_{s}-\varepsilon_{\infty }}{2}\right)}^{2}$$

where $\varepsilon_{s}$ is static permittivity and $\varepsilon_{\infty }$ is relative permittivity at the high-frequency limits. Each semicircle in the curve of $\varepsilon^{\prime }-\varepsilon^{\prime \prime }$ is generally denoted as the Debye or Cole–Cole semicircle. There are different numbers of distorted Cole–Cole semicircles belonging to S1–S3, and each semicircle corresponds to one Debye dipolar relaxation process. This reveals that carbon source content will influence the Debye relaxation process due to different matching between the $\varepsilon^{\prime }$ and $\varepsilon^{\prime \prime }$ values. These results well confirm that the dielectric loss in the 3D honeycomb-like FeNi_3_@C composites mainly comes from multiple-relaxations. It can be found more Cole–Cole semicircles of S2 and S3, compared to ones of S1. It is widely accepted that dielectric losses are not the only determinant in the evaluation of MA performances in ferrite-carbon composite absorbers [19]. Permeability and magnetic loss also need to be analyzed, which are the other crucial factors responsible for MA performances. The excellent performance of S2 benefits from the combined effect of dielectric loss and magnetic loss.

Figure 8d,e show the $\mu^{\prime }$ and $\mu^{\prime \prime }$ values for all the samples in the frequency range of 2.0–18.0 GHz. Figure 8d shows the $\mu^{\prime \prime }$ values for all the samples are in the range of 1.0–1.3 with some fluctuations. In particular, the S2 and S3 samples show obvious resonance peaks at different frequencies. The *μ*″ value of S2 is the largest among the three samples, and this indicates higher dissipation of magnetic energy. At the same time, the $\mu^{\prime \prime }$ values for S1 and S3 exhibit pretty similar trends. However, the $\mu^{\prime \prime }$ value for S3 becomes negative in the frequency range of 9.04–18.0 GHz. Generally speaking, the electromagnetic wave absorption materials consume magnetic energy of the incident EM wave, and thus, there are rarely negative $\mu^{\prime \prime }$ values [32]. However, negative values may result from the implicit Fabry–Perot resonance, porous structure of FeNi_3_@C, and Snoek limit at high frequencies [15,44,45,46]. According to the Maxwell equation, movement of electric charge first generates an alternating electric field and then forms a magnetic field. Moreover, the negative value of $\mu^{\prime \prime }$ indicates that the redundant magnetic energy that radiated out by the energy of the internal and external magnetic fields overloads the loss capability of FeNi_3_ nanocrystals [32,47]. The magnetic loss factors ($\tan\delta_{\mu }$) for all samples are presented in the Figure 8f, and the shape and tendency of $\tan\delta_{\mu }$ are similar to $\mu^{\prime \prime }$. In addition, $\tan\delta_{\mu }$ for S2 is higher than that for other samples, and this indicates that S2 has better magnetic loss capability. Interestingly, it is found that the enhanced MA performance of S2 is mainly caused by dielectric loss at 2–8.0 GHz, and magnetic loss plays a major role at high frequency, as seen in Figure 8c,f. It is known that, magnetic loss always results from natural ferromagnetic resonance, domain wall resonance, eddy current effects, and hysteresis [16]. Hysteresis loss can be ignored because of the weak field, and domain wall resonance loss can also be neglected because this resonance only occurs at a much lower frequency (MHz) [48,49]. Thus, the main loss mechanisms of absorbers are the eddy current effect and natural ferromagnetic resonance. The eddy current loss can be expressed as follows: $C_{0}=\mu^{\prime \prime }{\left(\mu^{\prime }\right)}^{-2}f^{-1}$. If the eddy current effect is the only factor that results in magnetic loss, the value of $C_{0}$ is nearly constant regardless of changes in frequency [16]. As seen in Figure 9d, the values of $\;C_{0}$ for all of the samples obviously fluctuate with an increase in frequency, and this reveals that the eddy current effect and natural ferromagnetic resonance both contribute to magnetic loss. As reported in many studies, there is a further increase in the resonance frequency because of the shape anisotropy that result from the small size effect of nanoparticles [49,50].

Nowadays, the preparation of FeNi_3_@C composites has been reported, and their MA properties have been investigated. Ding et al. [8] have reported FeNi_3_@C–paraffin composites containing 10 wt% for MA absorber with an RL of −47.6 dB. The absorption bandwidth was about 3.12 GHz in the range of 7.28–10.40 GHz for a layer of 3.0 mm thickness. Sun et al. [9] found FeNi_3_@C nanowires–paraffin composites containing 40 wt% FeNi_3_@C nanowires had an excellent MA performance. For a 2.0 mm thickness layer, an optimal RL of −43.3 dB was obtained and RL exceeding −10 dB was observed at 8.7–13.78 GHz. In our work, when the filler loading ratio of FeNi_3_@C/paraffin composites is only 30 wt%, the RL value can reach −40.6 dB at 10.04 GHz. Meanwhile, an ultra-wide absorption frequency bandwidth of 13.0 GHz (5.0–18.0 GHz over −10 dB) is obtained. Table 1 shows the MA properties of some reported FeNi_3_-based composites. Compared to recent reports, the 3D honeycomb-like FeNi_3_@C exhibits outstanding MA properties.

**Figure 10:**
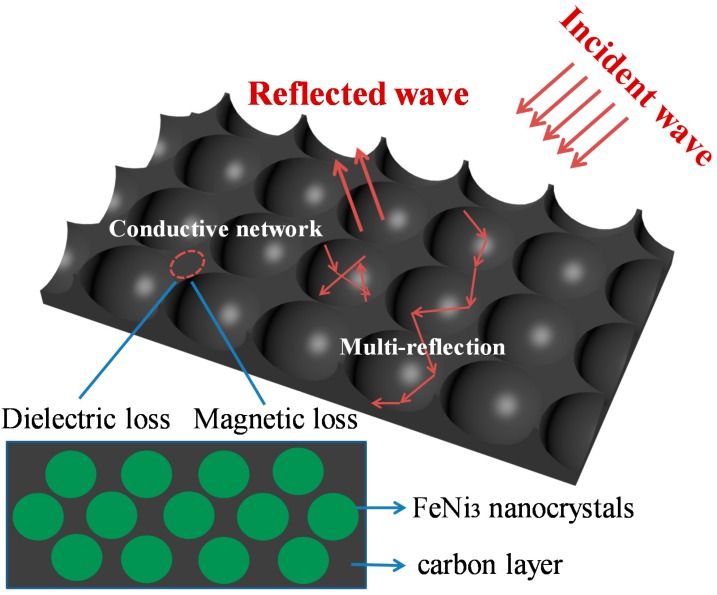
Schematic diagram of microwave absorption for the 3D honeycomb-like FeNi_3_@C.

Based on the above, the S2 sample has excellent MA properties and a broad bandwidth as a result of the synergistic effect between the dual loss (dielectric and magnetic loss) of the carbon layer and FeNi_3_ nanocrystals. However, beyond that, the unique structure also contributes to the improved MA properties. In Figure 10, when incident microwave penetrates into the absorbent, multiple reflections and scatterings occur in the 3D honeycomb-like structure, and this extends the propagation of the microwave pathway to largely attenuate the incident EM wave [4,10,22]. Dielectric loss is induced in dipole polarization and interfacial polarization, and these are respectively caused by a large number of defects and interfaces. Also, the 3D honeycomb-like structure forms a conductive network which is good for improving electrical loss. As expected, the 3D honeycomb-like FeNi_3_@C composites exhibit optimum MA performance.

## 4. Conclusions

In summary, 3D honeycomb-like FeNi_3_@C composites were successfully synthesized via a facile method that included drying and carbonization. During drying, metal ions evenly fill in the gaps of the PS spheres, and the template PS spheres are removed as a result of the formation of plentiful pores in the carbonization process. The 3D honeycomb-like FeNi_3_@C composites (S2) possess superb MA performances as expected. The optimal RL value is −40.6 dB at 3 mm coating thickness, and response absorption bandwidth (RL ≤ −10 dB) is 13.0 GHz when the thicknesses are only 2.0–4.5 mm. This means that 90% of the incident waves can be consumed by the absorber when the frequency is in the range of 5.0–18.0 GHz. The excellent MA performance can be ascribed to the combined effect of dual loss components, multiple interfacial polarizations, dipolar polarization, and the distinctive 3D honeycomb-like structure. Based on the above analyses and results, the 3D honeycomb-like FeNi_3_@C composites are promising for exploring new high-performance EM wave absorbing materials.

## Figures and Tables

**Table 1:** Some FeNi_3_-based composites for microwave absorption (MA) materials reported in publications.

Samples	Minimum RL Value (dB)	Frequency Range (RL ≤ −10 dB) GHz	Thickness (mm)	Content (wt%)	Reference
FeNi_3_/N-GN	−34.00	3.60–18.00	3.23	50	[4]
FeNi_3_/NiFe_2_O_4_	−15.00	unknown	1.40	80	[7]
FeNi_3_@C	−47.60	7.28–10.40	3.00	10	[8]
FeNi_3_@C	−43.30	8.70–13.78	2.00	40	[9]
RGO/FeNi_3_/Fe_3_O_4_	−46.60	6.10–15.36	1.90	60	[11]
FeNi_3_@RGO/MoS_2_	−30.39	unknown	2.00	40	[26]
FeNi_3_/epoxy	−20.00	13.10–16.20	1.60	unknown	[51]
S1	−36.43	5.40–18.00	4.00	30	This work
S2	−40.60	5.00–18.00	3.00	30	This work

## Supplementary Materials

The following are available online at https://www.mdpi.com/2079-4991/10/4/598/s1, Figure S1: SEM images of (a,b) S1, (c,d) S3. Figure S2: (a) Real parts of complex permittivity and (d) permeability, (b) imaginary parts of complex permittivity and (e) permeability, (c) dielectric and (f) magnetic loss tangents of S2 with the filler loadings of 10, 20, 30, and 40 wt%, respectively. Figure S3: The RL values of the S1–S3 with a thickness of 3.0 mm.
