# Supplementary material for: Ultrafine FeNi3 Nanocrystals Embedded in 3D Honeycomb-Like Carbon Matrix for High-Performance Microwave Absorption
Source: Nanomaterials (Basel). 2020 Mar 25;10(4):598. doi: 10.3390/nano10040598 (PMC7221889; doi:10.3390/nano10040598)
Supplement: Supplementary file 1 [file nanomaterials-10-00598-s001.pdf]

# Ultrafine $FeNi_3$ nanocrystals embedded in 3D honeycomb-like carbon matrix for high-performance microwave absorption

*Congai Han[1], Haiyan Zhang[1*], Danfeng Zhang[2], Yunfei Deng[1], Junyao Shen[1], Guoxun Zeng[1]*

*[1]School of Material and Energy, Guangdong University of Technology, Guangzhou 510006, China*

*[2] School of Computer Science and Technology, Guangdong University of Technology，Guangzhou, 510006,China*

*[*] Corresponding author. E-mail address: hyzhang@gdut.edu.cn*

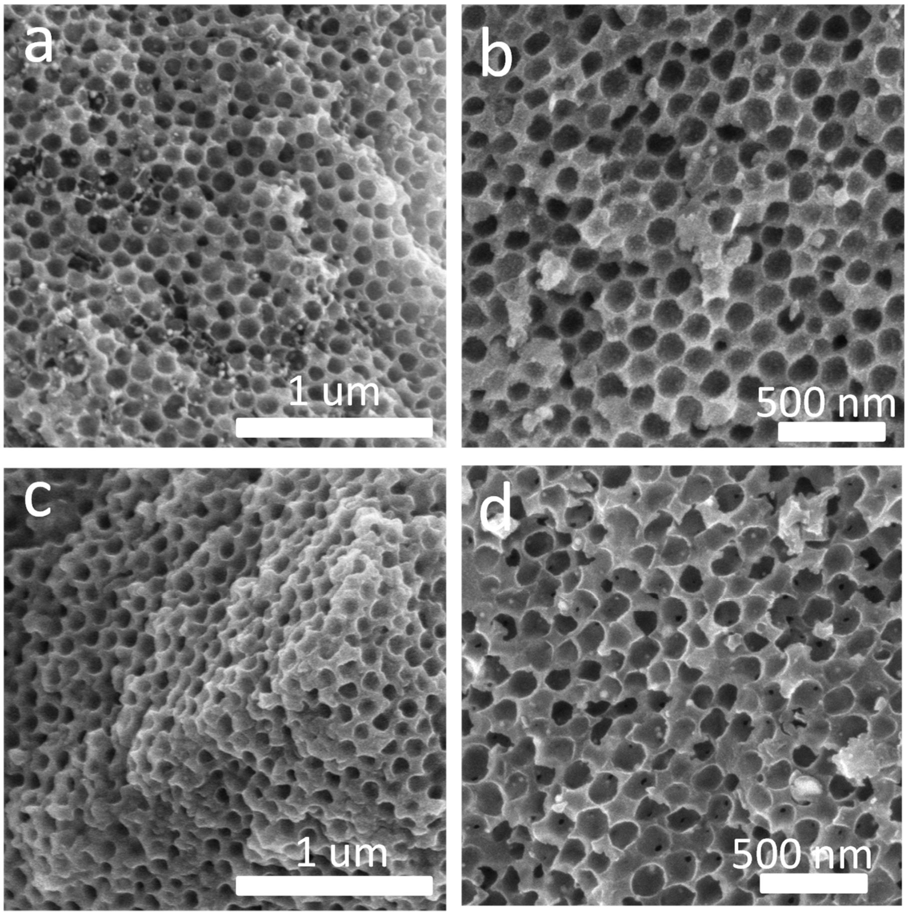


**Figure S1.** SEM images of (**a** and **b**) S1, (**c** and **d**) S3

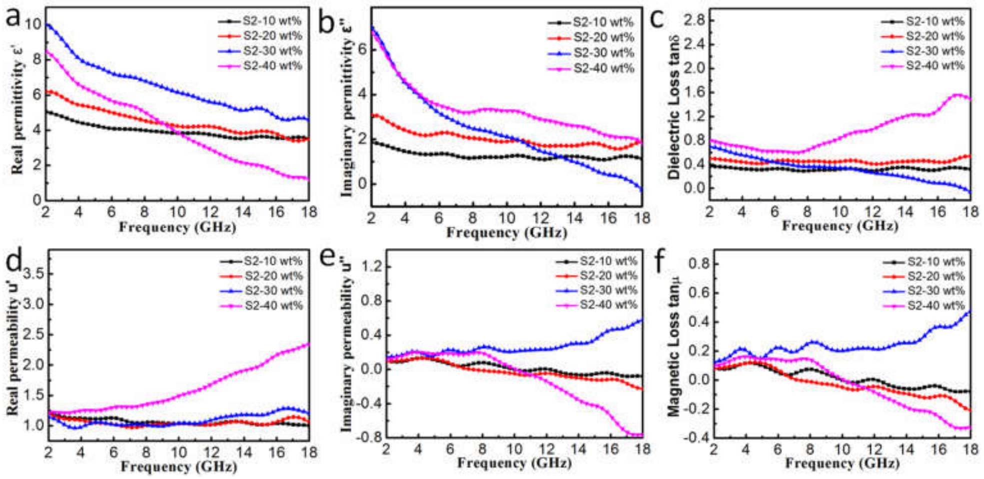


**Figure S2.** (**a**) Real parts of complex permittivity and (**d**) permeability, (**b**) imaginary parts of complex permittivity and (**e**) permeability, (**c**) dielectric and (**f**) magnetic loss tangents of S2 with the filler loadings of 10, 20, 30, and 40 wt%, respectively.

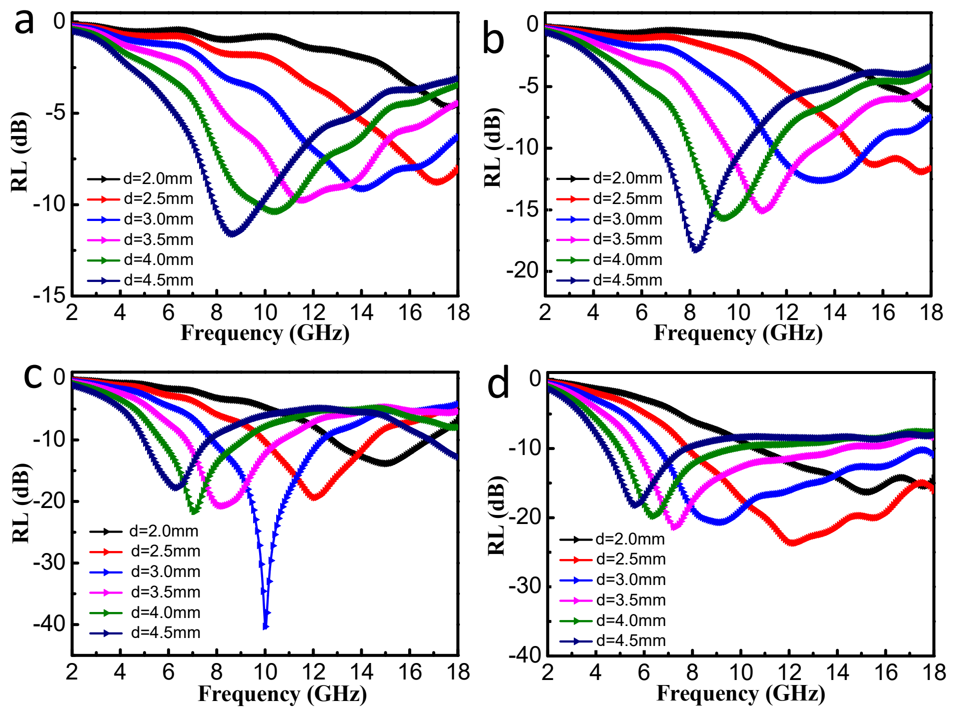


**Figure S3.** Calculated reflection loss (RL) curves of the S2–wax composites with different absorber thicknesses: (**a**) S2-10 wt%, (**b**) S2-20 wt%, (**c**) S2-30 wt%, and (**d**) S2-40 wt%.

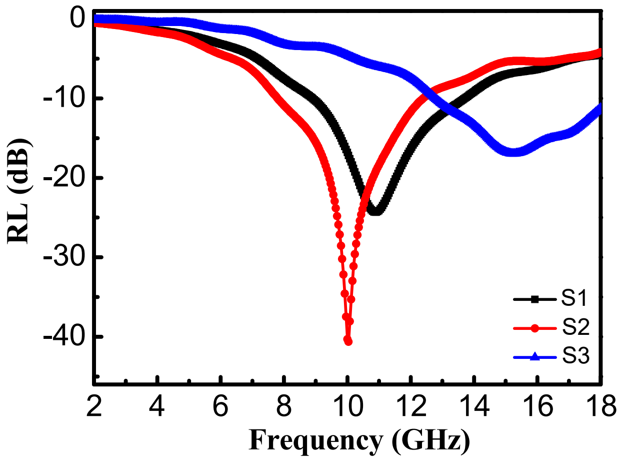


**Figure S4.** The RL values of the S1-S3 with a thickness of 3.0 mm.
